# Alterations of functional connectivity of the lateral habenula in subclinical depression and major depressive disorder

**DOI:** 10.1186/s12888-022-04221-6

**Published:** 2022-09-05

**Authors:** Lei Yang, Chaoyang Jin, Shouliang Qi, Yueyang Teng, Chen Li, Yudong Yao, Xiuhang Ruan, Xinhua Wei

**Affiliations:** 1 College of Medicine and Biological Information Engineering, Northeastern University, Shenyang, China; 2 Key Laboratory of Intelligent Computing in Medical Image, Ministry of Education, Northeastern University, Shenyang, China; 3 Department of Electrical and Computer Engineering, Stevens Institute of Technology, Hoboken, USA; 4 Department of Radiology, Guangzhou First People’s Hospital, School of Medicine, South China University of Technology, Guangzhou, China

**Keywords:** Subclinical depression, Major depressive disorder, Functional connectivity, Resting-state fMRI, Lateral habenula

## Abstract

**Background:**

Major depressive disorder (MDD) is a common cause of disability and morbidity, affecting about 10% of the population worldwide. Subclinical depression (SD) can be understood as a precursor of MDD, and therefore provides an MDD risk indicator. The pathogenesis of MDD and SD in humans is still unclear, and the current diagnosis lacks accurate biomarkers and gold standards.

**Methods:**

A total of 40 MDD, 34 SD, and 40 healthy control (HC) participants matched by age, gender, and education were included in this study. Resting-state functional magnetic resonance images (rs-fMRI) were used to analyze the functional connectivity (FC) of the posterior parietal thalamus (PPtha), which includes the lateral habenula, as the region of interest. Analysis of variance with the post hoc t-test test was performed to find significant differences in FC and clarify the variations in FC among the HC, SD, and MDD groups.

**Results:**

Increased FC was observed between PPtha and the left inferior temporal gyrus (ITG) for MDD versus SD, and between PPtha and the right ITG for SD versus HC. Conversely, decreased FC was observed between PPtha and the right middle temporal gyrus (MTG) for MDD versus SD and MDD versus HC. The FC between PPtha and the middle frontal gyrus (MFG) in SD was higher than that in MDD and HC. Compared with the HC group, the FC of PPtha-ITG (left and right) increased in both the SD and MDD groups, PPtha-MTG (right) decreased in both the SD and MDD groups and PPtha-MFG (right) increased in the SD group and decreased in the MDD group.

**Conclusion:**

Through analysis of FC measured by rs-fMRI, the altered FC between PPtha and several brain regions (right and left ITG, right MTG, and right MFG) has been identified in participants with SD and MDD. Different alterations in FC between PPtha and these regions were identified for patients with depression. These findings might provide insights into the potential pathophysiological mechanisms of SD and MDD, especially related to PPtha and the lateral habenula.

## Introduction

Major depressive disorder (MDD) is characterized by marked and persistent depression and is chronic, recurrent, and difficult to cure. Additionally, MDD is associated with a high suicide rate. These characteristics produce a severe adverse effect on the quality of life of patients with MDD [1–5].

Subclinical depression (SD) is regarded as an early stage indicator or precursor of MDD [6–8] because the depressive symptoms of patients with SD are not severe or persistent enough to be diagnosed as MDD [8–10]. SD is reported to affect 32% of Chinese college students [8, 11] and 23–39% of European college students [8, 12]. The public’s understanding of the mechanisms of MDD and SD is inadequate, and the diagnosis of MDD and SD still relies on physician interviews and scale tests, with a notable lack of accurate biomarkers.

Resting-state functional magnetic resonance imaging (rs-fMRI) examines the temporal correlation of spontaneous fluctuations in blood oxygen level dependent (BOLD) signals across brain regions [6, 13]. Functional connectivity (FC) measures the correlations between brain regions by calculating the Pearson correlation coefficient. By analyzing the FC between a specific brain region and other brain regions, abnormal connections can be identified. Exploring the FC changes between MDD and SD patients is of great significance [6, 13, 14]. Meta-analysis of rs-fMRI in MDD shows altered functional activity in a wide range of brain regions in the frontal-parietal network, frontal-limbic network, and default mode network [15, 16]. FC analyses of rs-fMRI data have characterized the altered brain network architecture based on intrinsic neural activity in the absence of any specified cognitive or affective load in MDD [17, 18].

Studies have shown that the lateral habenula (LHb) has an important relationship with the pathogenesis of depression [7, 19]. The lateral habenula (LHb) is a small epithelial structure located between the medial thalamus and the third ventricle [20–22], connecting the limbic system of the forebrain and the monoamine nucleus of the midbrain. According to the Montreal Neurological Institute (MNI) coordinates, the LHb is in the posterior parietal thalamus (PPtha) [7]. It is the anti-reward center of the brain and is considered to mediate most of the people's negative emotions, such as fear, tension, and anxiety. In depression, the LHb is involved in negative motivational value and decision-making [23–28]. A special discharge mode called cluster discharge of the LHb is a sufficient condition for the occurrence of depression [19, 29–31].

The LHb has become an anatomical target for testing antidepressants such as ketamine in animals and humans [19, 29, 32–35]. Concrete evidence demonstrates that bursts within the LHb drive depression in rats [19, 36–38]. Deep brain stimulation(DBS) of the LHb has been successfully used to treat patients with refractory MDD [39]. Additionally, clinical studies of DBS targeting the habenula in humans are underway [39–41]. Although this line of research is promising, it remains at an early stage and not all observations can be adequately explained by current theories on how LHb dysfunction contributes to depressive states [5]. Moreover, the connections of FC with the LHb are unknown in humans, especially when the FC is calculated from rs-fMRI data in both MDD and SD patients. Despite evidence that developmental trajectories in psychiatric disease are related to habenula volume [42], the FC alterations related to LHb in SD, MDD and health control (HC) groups have not been studied.

In this study, we compare the FC connected with LHb among MDD, SD and HC groups, and aim to clarify the FC alterations in HC, SD, and MDD. This study includes the collection of rs-fMRI data of three groups, extraction of the PPtha as the region of interest (ROI), calculation of the FC between PPtha and all other regions in the whole brain, and statistical analysis of altered FC.

## Materials and methods

### Participants

All participants in this study were college students from Guangzhou Medical University. A total of 40 MDD (11 males, 29 females), 34 SD (11 males, 23 females) and 40 HC (21 males, 19 females) subjects were screened from about 1000 college students over the period 2018–2019.

The MDD subjects were screened using the Hamilton Depression (HAMD) scale [43]. SD and HC subjects were screened using the Beck Depression Inventory II (BDI-II) scale [44]. MDD subjects had a HAMD score of > 17, SD subjects had a BDI-II score > 13 (total score, 14–28), and HC subjects had a BDI-II score of < 5 [44, 45]. The three groups were matched by gender, age, and education. All diagnostic criteria were following the *Diagnostic and Statistical Manual of Mental Disorders-IV* (DSM-IV) [46, 47]. Moreover, all participants were required to meet the following criteria: right handedness, no visible lesions on MRI, no neurological diseases, and no alcohol or drug dependence.

### MRI acquisition

MRI images were obtained using a 3-Tesla MRI scanner (Siemens, Erlangen, Germany). To control head movement and reduce MRI noise, foam pads and earplugs were used. The rs-fMRI images were obtained using an echo planar imaging sequence: echo time (TE) = 21 ms, repetition time (TR) = 2500 ms, flip angle (FA) = 90°, field of view (FOV) = 200 mm × 200 mm, matrix = 64 × 64, voxel size = 3.5 mm × 3.1 mm × 3.1 mm, and 42 slices with no gap. There were 200 time-points (or volumes) of rs-fMRI collected for each participant. The parameters of the T1-weighted images were as follows: TR = 2530 ms, TE = 2.34 ms, FA = 7°, FOV = 256 mm × 224 mm, and a slice thickness of 1.0 mm with no gap.

The participants were asked to stay relaxed and keep their eyes closed without sleeping. None of the participants showed significant structural damage based on routine MRI scans.

### Study design and main procedures

**Fig. 1:**
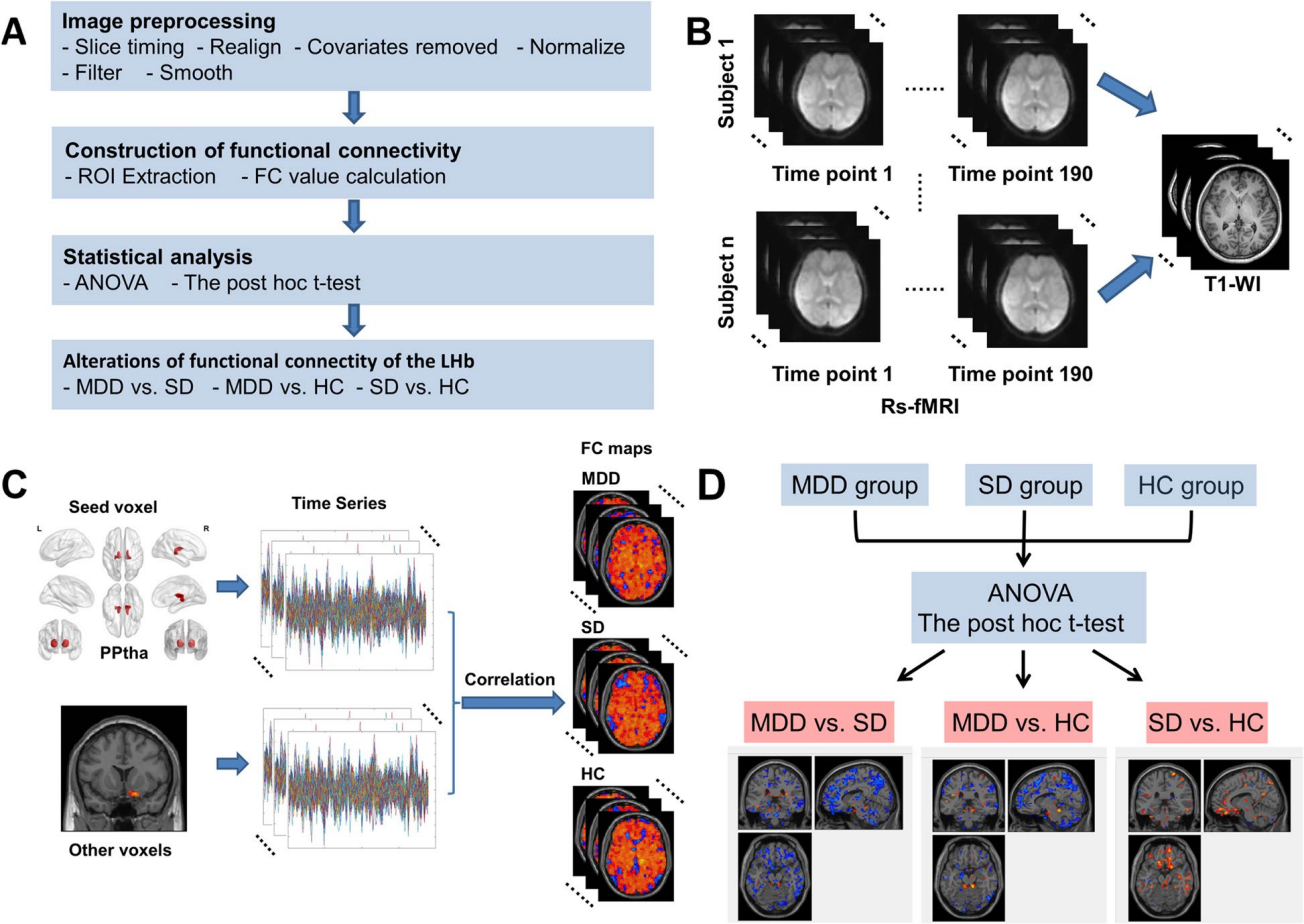
The study design and main procedures. **A** The overview of the study procedures; **B** Rs-fMRI image preprocessing; **C** The ROI extraction and FC calculation between PPtha and other brain regions; **D** The statistical analysis procedure of FC. (ROI, region of interest; FC, functional connectivity; ANOVA, analysis of variance; rs-fMRI, resting-state functional magnetic resonance imaging; T1-WI, T1-weighted imaging; PPtha, posterior parietal thalamus; R, right; L, left; MDD, major depressive disorder; SD, subclinical depression; HC, healthy control; vs., versus)

As shown in Fig. 1A, this study included four steps: (1) image preprocessing; (2) calculation of FC; (3) statistical analysis; (4) identification of alterations in FC of the LHb. In brief, the rs-fMRI image preprocessing was conducted and then the FC was calculated. One-way analysis of variance (ANOVA) with the post hoc t-test [48–50] was carried out during the statistical analysis. Then alterations in FC of the LHb were identified.

### Rs-fMRI image preprocessing

The rs-fMRI data were preprocessed using the Data Processing Assistant for Resting-State fMRI (DPARSF, http://rfmri.org/DPARSF) [51] based on Statistical Parametric Mapping (SPM12, http://www.fil.ion.ucl.ac.uk/spm). The substeps are given as follows (Fig. 1B):

1. To eliminate the unstable factors of MRI equipment and subjects, the first ten time points of each subject were removed.
2. The slice timing was corrected by using the middle slice as the reference.
3. Realignment was used to correct the head motion resulting from breathing, heartbeats, and uncontrolled factors during the scan. To control the head motion further, scrubbing was conducted for all rs-fMRI in image preprocessing. The framewise displacement (FD) type is “FD (Power)” [52], the FD threshold is 0.2 mm and each bad time point (FD > 0.2 mm) is removed as a separate regression value [52–54]. Finally, the patients with head motions > 2 mm and rotations > 2° were removed.
4. The T1 image was registered to the MNI space using the Diffeomorphic Anatomical Registration Through Exponentiated Lie Algebra (DARTEL) segmentation algorithm [55].
5. To control the head motion as little as possible, the nuisance covariates (i.e., Friston 24-parameter model, cerebrospinal fluid signal, and white matter signal) were regressed out.
6. The normalized T1 image parameters were used to match the functional image to the MNI space. The function images were resampled with a pixel size of 3 mm × 3 mm × 3 mm for normalization.
7. The images were filtered with the band-pass filter of 0.01–0.08 Hz.
8. Finally, a Gaussian kernel with a full-width at half maximum (FWHM) of 6 mm × 6 mm × 6 mm was used to smoothen the images.

### Calculation of FC

A seed-to-whole-brain method was used to calculate the FC (Fig. 1C). Specifically, the Brainnetome Atlas [56] with 246 subregions was used to locate the ROI. The left PPtha (239th subregion) and right PPtha (240th subregion) were combined to get the whole PPtha mask. The PPtha mask was selected as the seed and the FC between PPtha and other voxels in the whole brain was calculated.

The seed-based method in the FC module of the DPABI software ([57], http://www.rfmri.org/dpabi) was used to calculate the FC. First, the average time series of PPtha were calculated. Second, the time series of each voxel in the whole brain except the PPtha were extracted. The Pearson correlation coefficient between the PPtha and each voxel was obtained. The r values were converted to z-scores by DPABI to get the z-maps. Finally, the Anatomical Automatic Labeling (AAL) atlas was further utilized to identify where the clusters with significant differences were located.

### Statistical analysis

Demographic data from the MDD, SD, and HC groups were compared using the Statistical Package for the Social Sciences software, version 24.0 (SPSS, https://www.ibm.com/products/spss-statistics). One-way ANOVA analysis was performed to assess differences in age and educational level, and a chi-squared test was performed to assess gender differences.

The presence of significant age differences, root mean squared (RMS) framewise displacement (FD) (one-way ANOVA tests, *p* < 0.05), gender (chi-squared test, *p* < 0.05), and educational level (one-way ANOVA tests, *p* < 0.05) among the MDD, SD, and HC groups was tested. The difference in BDI-II score between the SD and HC groups was tested using a two-sample t-test; *p* < 0.001 indicates a significant statistical difference.

Figure 1D illustrates the statistical analysis procedure. The statistical analysis was conducted by DPABI software. The mean framewise displacement (i.e., Mean FD_Jenkinson) was taken as the covariate to control the impact of unnecessary head motion in the statistical analysis. One-way ANOVA (*p* < 0.05, Bonferroni corrected) in the statistical analysis module of DPABI software was performed to compare variables among the three groups (HC, SD, and MDD). A mask was built according to the results of ANOVA. Based on the mask, the inter-group differences were obtained by using the post hoc t-test. Two-tailed Gaussian random field (GRF) correction [58] (voxel threshold of *p* < 0.005 and cluster threshold of *p* < 0.05, and voxel threshold of *p* < 0.001 and cluster threshold of *p* < 0.05) was performed during the two-sample t-test (the post hoc test). Finally, the brain regions with significant FC connected with PPtha were identified.

## Results

### Demographic and clinical characteristics

A total of 40 MDD subjects, 34 SD subjects, and 40 HC subjects participated in the rs-fMRI process. Using the head motion standard of 2 mm translation and 2° rotation, 2 MDD subjects, 8 SD subjects, and 7 HC subjects were excluded. Finally, the data from 38 MDD subjects, 26 SD subjects, and 33 HC subjects were included in the statistical analysis. Demographic and clinical data are presented in Table 1. No significant differences in terms of age, gender, and educational level (*p* > 0.05) were found among the MDD, SD, and HC groups. All participants with MDD and SD showed higher symptom scores than those in the HC group (*p* < 0.001).

**Table 1:** Demographic and clinical characteristic of three groups of participants

Characteristics	MDD (*n* = 38)	SD (*n* = 26)	HC (*n* = 33)	*p*-value
Gender, female/male	27/11	16/10	16/17	0.150^a^
Age, years	21.13 ± 6.17	19.65 ± 1.77	19.24 ± 0.94	0.120^b^
Education, years	12.94 ± 2.60	13.36 ± 0.92	13.18 ± 0.87	0.492^b^
BDI-II score	-	22.46 ± 7.73	1.55 ± 1.44	< 0.001^c^
HAMD score	21.51 ± 4.58	-	-	-
RMS FD	0.45 ± 0.02	0.54 ± 0.06	0.52 ± 0.06	0.177^b^

Abbreviations: *MDD* major depressive disorder, *SD* subclinical depression, *HC* healthy control, *BDI-II* Baker Depression Inventory-II, *HAMD* Hamilton Depression, *RMS FD* root mean squared framewise displacement

^a^*p* value obtained by chi-squared test

^b^*p* values obtained by one-way analysis of variance tests

^c^*p* value obtained by two-sample t-test

The RMS FD for each participant were calculated and compared across groups using one-way ANOVA tests. The results presented in Table 1, do not show any significant differences in RMS FD among the three groups.

### ANOVA results for MDD, SD, and HC groups

**Fig. 2:**
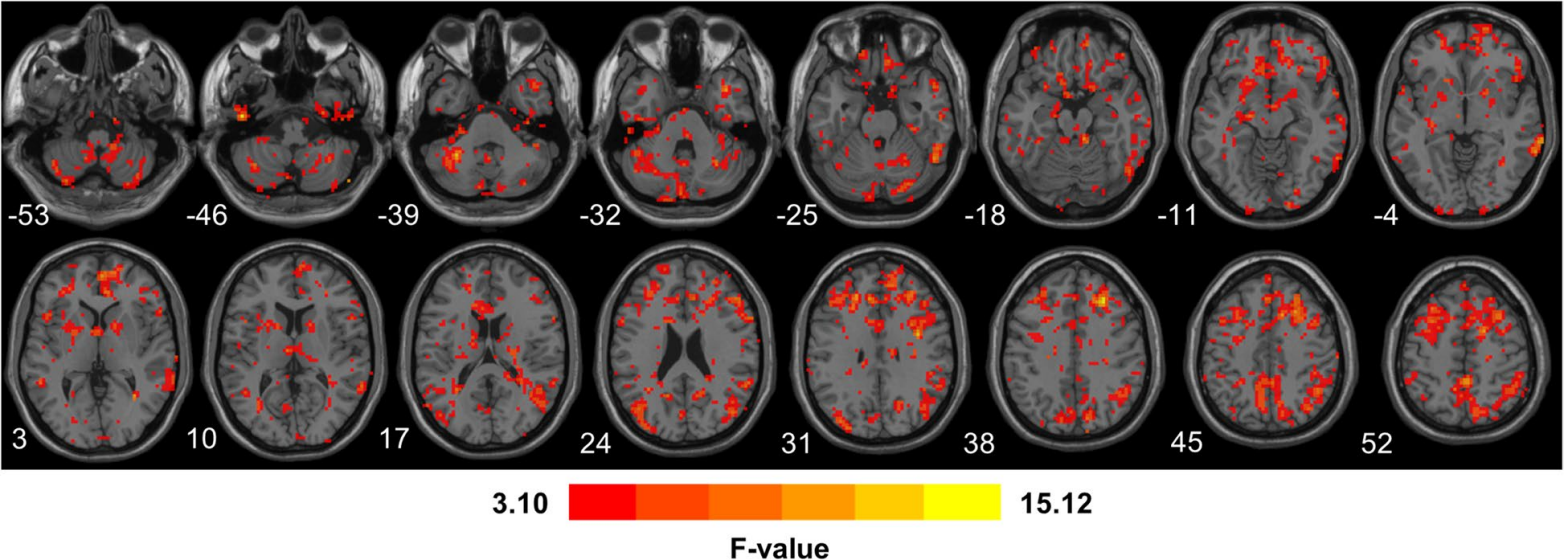
Brain regions with significant differences in functional connectivity connected with the PPtha among three groups (*p* < 0.05, *z* > 3.1) after ANOVA analysis

Table 2 and Fig. 2 show the one-way ANOVA analysis of the FC among the three groups. Significant group differences in FC were observed in Temporal_Inf_L (AAL), Superior Temporal Gyrus, Temporal_Pole_Mid_R (AAL), Middle Frontal Gyrus, Frontal_Sup_R (AAL), Medial Frontal Gyrus, Cerebellum Posterior Lobe, Cerebellum Anterior Lobe, Middle Temporal Gyrus, Precentral Gyrus, Supp_Motor_Area_R (AAL), Precuneus_R (AAL), Postcentral Gyrus, and Parietal_Sup_L (AAL).

**Table 2:** Brain regions with significantly different FC connected with the posterior parietal thalamus among the SD, MDD patients, and healthy controls

Side	Region	MNI coordinate (x, y, z)			Peak intensity	Voxels
L	Temporal_Inf_L (AAL)	-48	-15	-45	14.4219	53
R	Superior Temporal Gyrus Temporal_Pole_Mid_R (AAL)	39	12	-36	10.6047	107
R	Middle Frontal Gyrus	27	30	39	14.3868	2733
R	Frontal_Sup_R (AAL)Medial Frontal Gyrus Medial Frontal Gyrus	9	45	30	8.3849	98
R	Cerebellum Posterior Lobe	27	-78	-24	7.2024	56
R	Cerebellum Anterior Lobe	-30	-51	-39	11.687	589
R	Middle Temporal Gyrus	66	-48	-6	15.1143	2345
R	Precentral Gyrus	21	-27	60	5.7781	33
R	Supp_Motor_Area_R (AAL) Precuneus_R (AAL)	30	-66	6	9.4220	193
R	Temporal_Inf_R (AAL)	27	-12	-45	7.8717	80
R	Postcentral Gyrus	-48	-36	57	5.9806	43
L	Parietal_Sup_L (AAL)	-30	-66	6	8.3664	193

Abbreviations: *MNI* Montreal Neurological Institute, *R* right, *L* left, *AAL* Anatomical Automatic Labeling

### Altered FC connected with PPtha for MDD versus SD

**Fig. 3:**
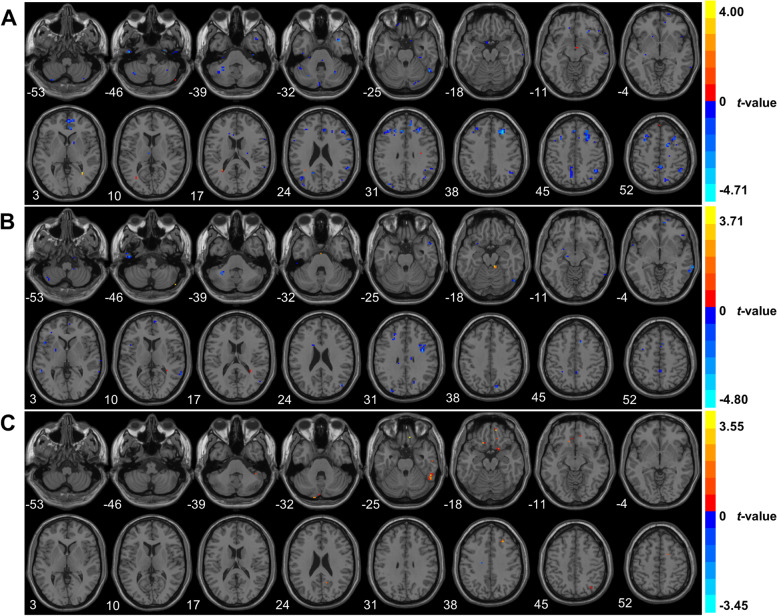
Brain regions with significant differences in functional connectivity connected with the posterior parietal thalamus among three groups (cluster *p* < 0.05, *z* > 2.8). Color bars represent the t-value from group analysis. **A** MDD vs. SD. **B** MDD vs. HC. **C** SD vs. HC

The significantly different FC between the PPtha and other brain regions in the MDD and SD groups is shown in Table 3 and Fig. 3(A). The MDD group presents the significantly increased FC between the PPtha and left inferior temporal gyrus (ITG) (Temporal_Inf_L [AAL]) in comparison with SD. Moreover, the FC between the PPtha and right superior temporal gyrus (STG), middle temporal gyrus (MTG), middle frontal gyrus (MFG), dorsolateral superior frontal gyrus (Frontal_Sup_R [AAL]), and medial frontal gyrus is decreased in the MDD group.

**Table 3:** Brain regions with significantly different FC connected with the posterior parietal thalamus

Comparison		Side	Region	MNI coordinate (x, y, z)			T value	Voxels(voxel *p* < 0.005)	Voxels(voxel *p* < 0.001)
MDD vs. SD	MDD > SD	L	Temporal_Inf_L (AAL)	-42	-36	-15	3.8092	1	1
	MDD < SD	R	Superior Temporal Gyrus Temporal_Pole_Mid_R (AAL)	39	12	-36	-4.0310	17	9
		R	Middle Frontal Gyrus	27	33	39	-4.7146	108	56
		R	Frontal_Sup_R (AAL) Medial Frontal Gyrus	18	3	54	-4.1943	33	10
MDD vs. HC	MDD > HC	R	Cerebellum Posterior Lobe	51	-75	-45	3.5093	3	1
		R	Cerebellum Anterior Lobe	9	-36	-18	3.7061	7	2
	MDD < HC	R	Middle Temporal Gyrus	66	-48	-6	-4.8021	52	28
		R	Precentral Gyrus	36	0	30	-4.3604	18	3
		R	Supp_Motor_Area_R (AAL) Precuneus_R (AAL)	3	-42	51	-3.1291	10	3
SD vs. HC	SD > HC	R	Temporal_Inf_R (AAL)	51	-48	-27	3.5549	18	5
		R	Middle Frontal Gyrus	27	30	39	3.4004	8	1
		R	Postcentral Gyrus	24	-33	75	3.3121	4	1
	SD < HC	L	Parietal_Sup_L (AAL)	-36	-54	66	-3.4485	6	3

Abbreviations: *MDD* major depressive disorder, *SD* subclinical depression; *HC* health control, *MNI* Montreal Neurological Institute, *T value*, t-statistical value of peak voxel showing, *R* right, *L* left, *AAL* Anatomical Automatic Labeling

### Altered FC connected with PPtha for MDD versus HC

In the MDD group, the FC between the PPtha and right cerebellum posterior lobe and cerebellum anterior lobe is higher than in the HC group. However, in comparison with HC, the FC between the PPtha and right MTG, precentral gyrus, Supp_Motor_Area_R [AAL], and precuneus is lower in the MDD group. Detailed results are shown in Table 3 and Fig. 3B.

### Altered FC connected with PPtha for SD versus HC

The FC between the PPtha and right ITG (Temporal_Inf_R [AAL]), MFG, and postcentral gyrus is higher in the SD group than that in the HC group. Conversely, the FC between the PPtha and left superior parietal gyrus (SPG, Parietal_Sup_L [AAL]) is lower, as shown in Table 3 and Fig. 3C.

### Important FC alteration for HC, SD and MDD

**Fig. 4:**
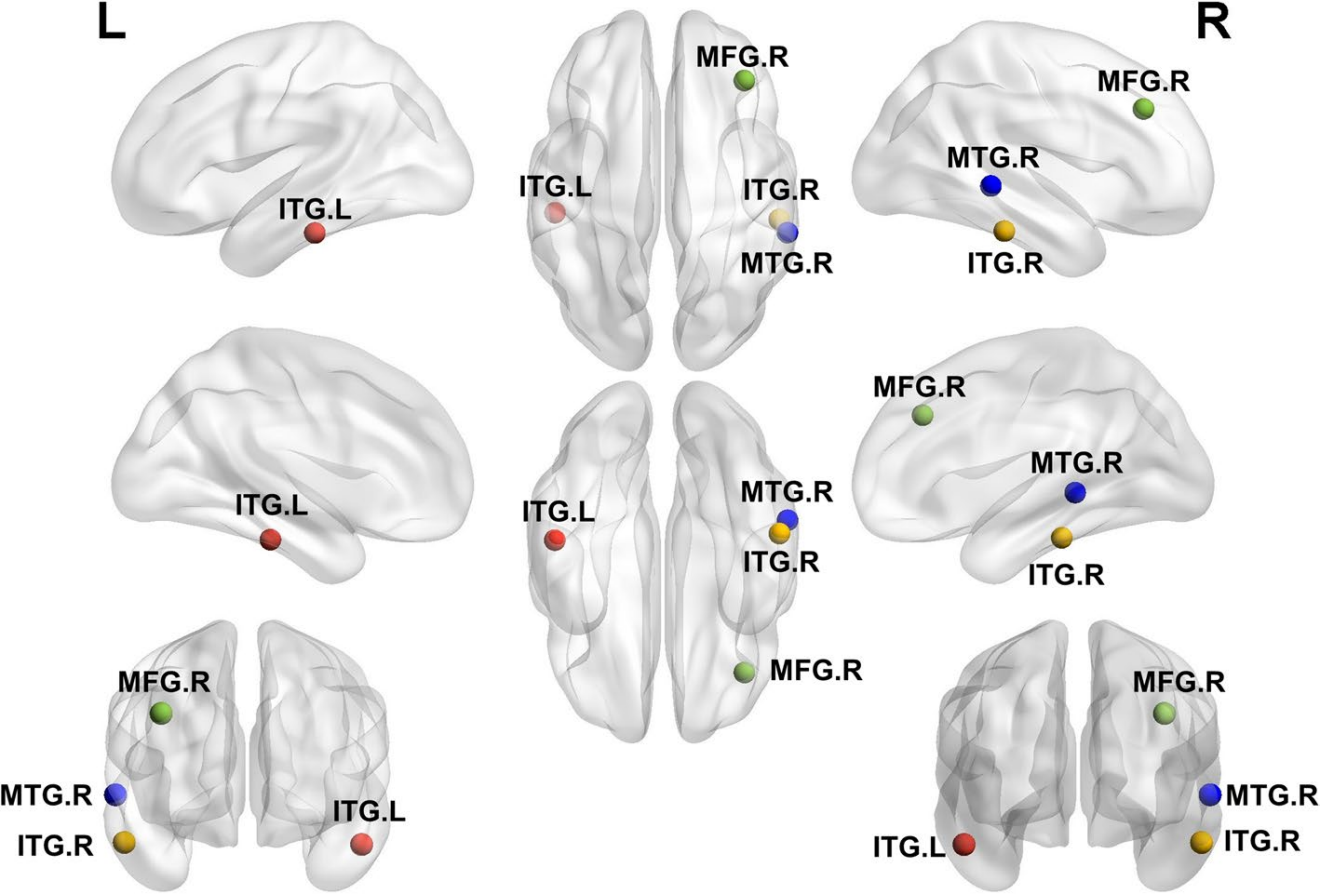
Four main brain regions characterizing the alterations of functional connectivity connected with the posterior parietal thalamus in HC, SD, and MDD groups (ITG.L, the left inferior temporal gyrus; ITG.R, the right inferior temporal gyrus; MTG.R, the middle frontal gyrus; MFG.R, the right middle temporal gyrus)

**Fig. 5:**
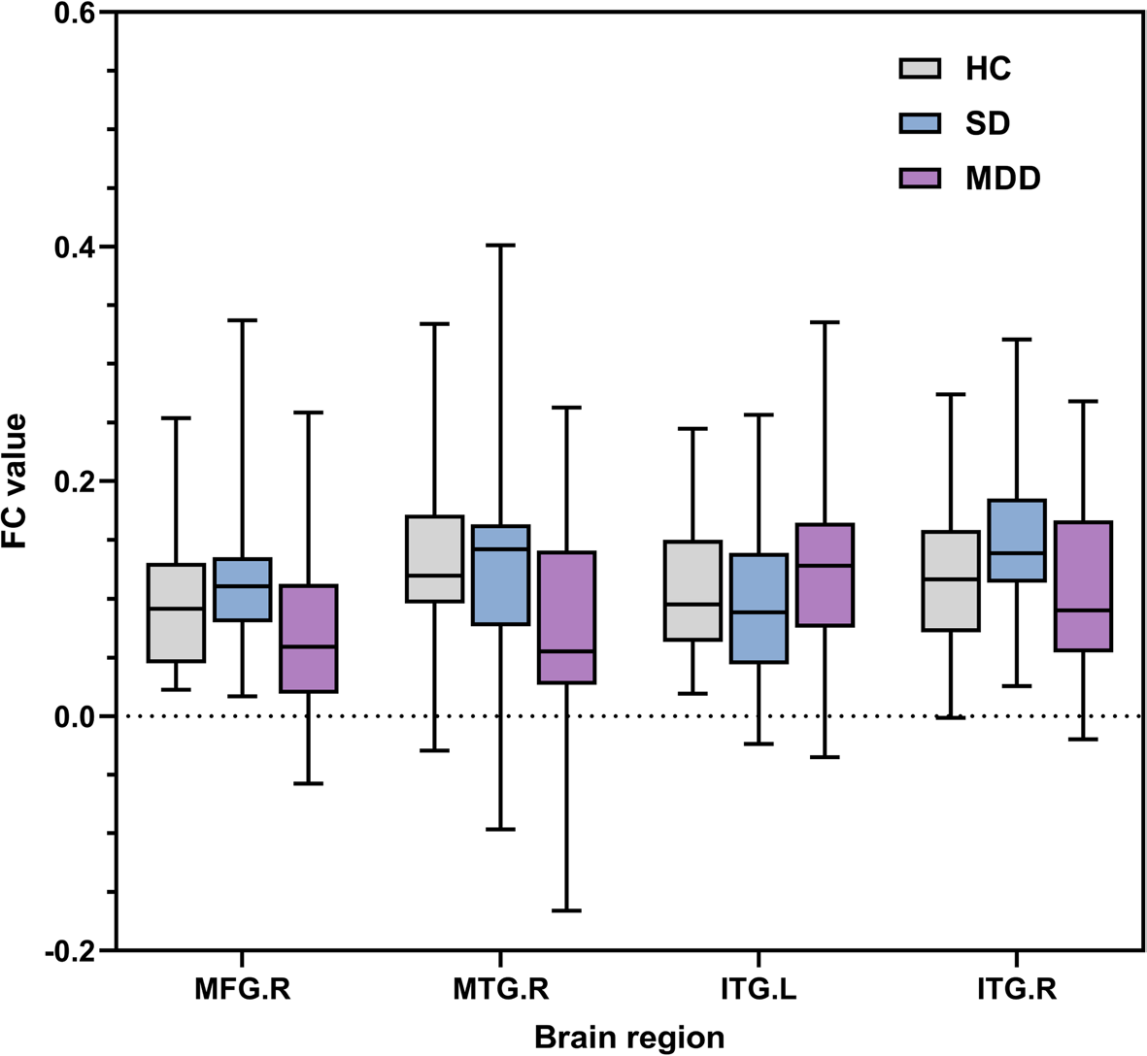
The FC value for the significant ROIs for each group (ITG.L, the left inferior temporal gyrus; ITG.R, the right inferior temporal gyrus; MTG.R, the middle frontal gyrus; MFG.R, the right middle temporal gyrus)

By comparing the FC alterations in MDD vs. SD, MDD vs. HC and SD vs. HC, the potential tendency for depression have been identified. Specifically, increased FC was found in ITG in the comparison of MDD vs. SD (left side) and SD vs. HC (right side). Conversely, decreased FC of the MDD group was observed between the PPtha and right MTG in the comparison of MDD vs. SD and MDD vs. HC. Additionally, the FC between the PPtha and right MFG was higher in the SD group than in both the MDD and HC groups. The locations of the four identified regions, (left ITG, right ITG, right MFG, and right MTG) are shown in Fig. 4. The FC value for MFG.R, MTG.R, ITG.L and ITG.R for each group is shown in Fig. 5.

It should be noted that GRF correct method was used with two criteria: (1) voxel *p* < 0.005 and cluster *p* < 0.05; (2) voxel *p* < 0.001 and cluster *p* < 0.05. As shown in Table 3, the brain regions with significant differences were the same in the two cases. The voxel size of clusters with significant differences was small (e.g., 1 voxel in some cases) while voxel *p* < 0.001 and cluster *p* < 0.05, potentially suggesting that the correct standard was too strict.

## Discussion

To the best of our knowledge, this is the first study comparing the FC of PPtha among patients with MDD, SD, and HC. Altered FC between PPtha and several regions was identified in all three pairwise group comparisons. The regions included the ITG, MTG, and MFG. The FC alteration tendency from HC to SD and MDD was identified by comparing the three groups. These findings provide a new perspective and thinking for exploring the pathophysiological mechanism of depression. The significance of these findings is discussed in the following subsections.

### Importance of LHb and PPtha in depression

SD has been considered the precursor to MDD. The cluster discharge of the LHb in rats is sufficient for the occurrence of depression [19, 29, 31]. Undoubtedly, the FC between the LHb and other related regions is affected. The LHb is an important subregion of the PPtha. In this study, PPtha was chosen as the ROI for FC analysis to research which region the LHb influenced. We have used two steps to ensure the habenula is located within the PPtha ROI for all participants. First, according to the reported locations of the habenula and PPtha in MNI coordinates, we can know that the habenula is located in the PPtha region. In our previous study [7], the habenula was located in the PPtha region in the same way as used in the current study, and abnormal brain connections with PPtha had been identified. Second, after determining the PPtha ROI, one experienced neurologist confirmed that the habenula was located within the PPtha ROI for each participant via visual inspection of T1-W images and prior anatomical knowledge.

The PPtha in the Brainnetome Atlas was used as the ROI, instead of LHb, because the precise location of the LHb is difficult to determine. First, the small volume of the LHb was a limitation in some studies. The habenula volume in vivo is estimated to be closer to 18.5 mm^3^ per hemisphere based on structural MRI and postmortem measurements [59, 60]. This may be an underestimation due to the difficulty of resolving the lateral and anterior boundaries of the habenula. The LHb volume is smaller than 18.5 mm^3^ per hemisphere, which can be smaller than the voxel size of standard fMRI [20, 59, 61]. Second, 3 T fMRI with whole-brain coverage and appropriate repetition times could only be reliably achieved with a resolution ≥ 27mm^3^. In this case, the conventional fMRI voxel size is larger than the LHb. Finally, the smoothing kernels with 5–12 mm FWHM are larger than the LHb. The habenula signal is likely to be contaminated by adjacent structures, such as the medial dorsal thalamus or the epithalamic paraventricular nucleus [20].

### Increased FC between PPtha and ITG in both MDD and SD

All three pairwise group comparisons showed abnormal FC between the PPtha and the temporal lobe. Specifically, the FC between the PPtha and ITG in the MDD group was higher than that in the SD group. FC in the SD group was higher than that in the HC group. It is speculated that the FC between the PPtha and ITG is gradually enhanced during the progression from HC to MDD.

### Decreased FC between PPtha and MTG in MDD

The FC between the PPtha and right MTG was lower in the MDD group than that in the SD and HC groups. It is speculated that the FC between the PPtha and right MTG gradually decreases as depression progresses. One study reported a decreased connectivity between the prefrontal thalamus and the MTG in MDD patients [6], which may provide a basis for estimating the development stage of depression. Comparing the SD and HC groups, no significant differences in FC were found between the PPtha and MTG. This finding might suggest that the PPtha-MTG connection abnormality is common and consistent in both the SD and MDD groups.

The ITG and the MTG are crucial regions in the temporal cortex. Some research has reported that thalamo-temporal connectivity changes significantly in MDD patients [62]. Moreover, higher thalamo-temporal connectivity is associated with more severe depressive symptoms, suggesting an association with the core psychopathology of MDD [62, 63]. Numerous PET and fMRI studies have shown that some lateral parts of the temporal cortex play significant roles in aspects of affective processing and social cognition [64]. Other studies have considered that the overall function of the anterior temporal lobe may be the semantic processing, which is personally, socially, and emotionally relevant [65]. Additionally, modest evidence suggests the presence of small clusters of decreased connectivity between the frontal cortex and medial thalamus [62]. Our findings are consistent with those of other neuroimaging studies, and possibly reflect the emotional and somatosensory dysregulation in SD and MDD patients.

### Abnormal FC between PPtha and MFG in SD

In the MDD and SD groups, and the SD and HC groups, the FC between the PPtha and frontal lobes showed an abnormal value. The FC between the PPtha and right MFG in the MDD group was lower than that in the SD group. Moreover, the FC in the SD group was higher than that in the HC group. The FC between the hippocampus and MFG was impaired in MDD patients [66, 67]. Moreover, altered FC between the hippocampus and MFG has been reported in older adults with SD [68]. The left MFG indicates increased spontaneous neural activity in SD patients [8]. MFG is involved in regulating the intensity of response to emotional stimuli, and changes in MFG functional activities may lead to inappropriate responses to emotional events [69]. The depressive symptoms occur as a result.

Notably, the MFG is a crucial part of the dorsolateral prefrontal cortex (DLPFC), which has been tightly linked to depression [8]. In depression, DLPFC is involved in mood regulation. The DLPFC activity is inhibited at rest but increases when symptoms are relieved [70, 71]. Most importantly, regions of the DLPFC have been the targets for repetitive transcranial magnetic stimulation (rTMS) in depression treatment [7, 72, 73].

The mechanism underlying the higher PPtha-MFG connection in the SD group compared with both the MDD and HC groups is unknown. Here, we speculate that the connection between the PPtha and MFG may be severely changed in MDD patients. Another possibility is that the MFG or the PPtha may have been impaired in the post-depression stage.

### Limitations and future works

There are several limitations in this study. First, although this study included three groups of rs-fMRI data, the sample size is largely small. This factor limited the statistical significance. Second, we didn’t extract the accurate LHb as the ROI but used the PPtha region from the Brainnetome Atlas. This change may affect our findings. This study investigated the FC between the PPtha and ITG, MTG, and MFG for MDD and SD. The validity of our findings requires verification through further studies.

In future work, the sample size will be expanded. Subjects from the WU-Minn Human Connectome Project (HCP) Consortium’s 500 Subjects Release can be included [60, 71]. Furthermore, 246 × 246 matrices of functional brain networks can be constructed using the human Brainnetome Atlas [56]. The information of each node and its connections can be further analyzed from the viewpoint of brain networks. Finally, after calculating the FC connections among different brain regions, machine learning models could be constructed to classify the MDD and SD participants [74].

## Conclusion

Through analyzing the FC measured by using rs-fMRI data, this study explored the altered FC between PPtha and other brain regions in SD and MDD subjects. The FC between the PPtha and ITG, right MTG, and right MFG showed abnormality in both MDD and SD subjects. Different alterations for FC between PPtha and these brain regions were present for HC, SD, and MDD: increased PPtha-ITG (left and right) FC; decreased PPtha-MTG (right) FC; initially increased and subsequently decreased PPtha-MFG (right). These findings might provide insight into potential pathophysiologic mechanisms of SD and MDD, especially related to the lateral habenula.

## Data Availability

The datasets used and/or analysed during the current study are available from the corresponding author on reasonable request.

## Abbreviations

AAL: Anatomical automatic labeling
ANOVA: Analysis of variance
BDI-II: Beck Depression Inventory-II
BOLD: Blood oxygen level dependent
DARTEL: Diffeomorphic Anatomical Registration Through Exponentiated Lie Algebra
DBS: Deep brain stimulation
DLPFC: Dorsolateral prefrontal cortex
DPARSF: Data processing assistant for resting-state fMRI
DSM-IV: Diagnostic and Statistical Manual of Mental Disorders-IV
EPI: Echo planar imaging
FA: Flip angle
FC: Functional connectivity
FOV: Field of view
FWHM: Full-width at half maximum
GRF: Gaussian random field
HAMD: Hamilton Depression
HC: Healthy control
ITG: Inferior temporal gyrus
LHb: Lateral habenula
MDD: Major depressive disorder
MFG: Middle frontal gyrus
MNI: Montreal neurological institute
MTG: Middle temporal gyrus
PPtha: Posterior parietal thalamus
ROI: Region of interest
rs-fMRI: Resting-state functional magnetic resonance imaging
rTMS: Repetitive transcranial magnetic stimulation
SD: Subclinical depression
SPM: Statistical Parametric Mapping
SPSS: Statistical package for the social sciences
TE: Echo time
TR: Repetition time
